# Spatial robustness of P100 latency compared with amplitude: a data-driven rationale for visual pathway monitoring during neuroendovascular procedures

**DOI:** 10.3389/fnins.2026.1956999

**Published:** 2026-09-30

**Authors:** Su Ji Lee, Sung-Chul Jin, Sung-Tae Kim, Hee-Eun Choi, Sang-Ku Park, Minjae Cho

**Affiliations:** 1 Department and Research Institute of Rehabilitation Medicine, Yonsei University College of Medicine, Seoul, Republic of Korea; 2 Department of Neurosurgery, Inje University Haeundae Paik Hospital, Busan, Republic of Korea; 3 Department of Rehabilitation Medicine, Inje University Haeundae Paik Hospital, Busan, Republic of Korea; 4 Department of Neurosurgery, Konkuk University Medical Center, Seoul, Republic of Korea; 5 Department of Neurosurgery, Seoul National University Bundang Hospital (SNUBH), Seongnam-si, Gyeonggi-do, Republic of Korea; 6 Department of Neurosurgery, Daegu Good Morning Hospital, Daegu, Republic of Korea

**Keywords:** intraoperative neurophysiological monitoring, neuroendovascular procedures, P100 amplitude, P100 latency, spatial variability, visual evoked potential

## Abstract

Intraoperative visual evoked potential (VEP) monitoring has a reputation for being unreliable, and much of that belongs to P100 amplitude. Amplitude shifts with electrode position, anesthetic depth, and dipole geometry. Practice has nonetheless tended to lean on amplitude, and to treat latency as the weaker of the two. Neuroendovascular procedures give reason to shift that emphasis. During open surgery there can be a mixture of direct and ischemic neural injury, whereas in an endovascular procedure the brain is not dissected and the monitored pathway is threatened almost exclusively through perfusion. Oligodendrocytes are more vulnerable to ischemia than neurons, so myelinated conduction may slow before neurons are lost, and latency may prolong before amplitude falls. We first asked whether latency can be measured reliably enough. In a retrospective series of 49 eyes from 28 patients, the median spatial variability index across O1, Oz, and O2 was 2.10% for P100 latency and 40.9% for amplitude. Amplitude varied more than latency in 47 of 49 eyes and in all 28 patients. Latency behaves as a stable temporal measure of signal arrival, amplitude as a spatially sampled field measure. Some evidence for this exists in intraprocedural somatosensory monitoring. In an exploratory analysis of successfully reperfused thrombectomy patients, tree-based models separated 90-day functional outcome more consistently and efficiently by latency than by amplitude. If VEP latency behaves the same way, monitoring need not depend on the parameter that is hardest to trust, and VEP could be used actively in these procedures. We therefore propose that reproducible P100 latency prolongation may mark evolving visual pathway ischemia, and specify three studies that would test it. The first measures the temporal variability of intraoperative P100 latency and reports the smallest change it can resolve. That number sets the floor for any alarm threshold, including one below the conventional 10%. The second follows retrochiasmal perfusion-risk procedures with concurrent electroretinography, asking whether latency prolongation tracks angiographic events and precedes major amplitude loss. The third separates retinal from central contributions in ophthalmic-territory procedures. The present data establish spatial robustness only. Temporal stability and ischemia detection are the endpoints of those studies.

## 1 Introduction

Intraoperative visual evoked potential (VEP) monitoring is used to detect functional compromise of the visual pathway (Thirumala et al., 2011; Sato et al., 2024). It has a reputation for being less dependable than somatosensory or motor monitoring, and no consensus alarm criterion exists for it. Between the two available parameters, practice leans on amplitude. An alarm usually rests on a reduction in P100 amplitude or a loss of the waveform (Jashek-Ahmed et al., 2021; Nakagawa et al., 2022; Tao et al., 2024). Latency is widely regarded as the less reliable of the two (Jashek-Ahmed et al., 2021), although a combined amplitude–latency criterion has been proposed (Kodama and Goto, 2020). Amplitude, however, carries its own difficulty. Electrode position, stimulation quality, anesthetic depth, cortical dipole orientation, background noise, and waveform morphology all influence its value (Hayashi and Kawaguchi, 2017; Šuštar Habjan et al., 2025). A given amplitude change may reflect true visual pathway dysfunction, or it may reflect technical and physiologic fluctuation. This ambiguity limits the confidence with which a single amplitude change can be acted upon during a procedure (Mattogno et al., 2023).

Warning criteria for evoked potential monitoring were developed mainly in open surgery, and they use both parameters. The conventional somatosensory alarm is a 50% decrease in amplitude and/or a 10% increase in latency (Toleikis et al., 2024). Open surgery, however, is not one setting. When a lesion is separated from the surrounding brain, the pathway is threatened by traction, compression, coagulation heat, and direct manipulation. When an aneurysm is clipped or a carotid artery is opened, a vessel is clamped, and ischemia becomes a threat as well. Somatosensory monitoring is used in both, and these vascular operations are among its established indications (Toleikis et al., 2024). Where both kinds of threat occur, a criterion that weighs both parameters is the right one.

Neuroendovascular procedures sit at one end of this range. The brain is not dissected, and the pathway is threatened almost entirely through blood flow. The mechanisms are temporary or permanent occlusion, distal embolization, perforator compromise, vasospasm, flow reduction, and incomplete reperfusion (Habtezghi et al., 2023). Even the complications that do injure tissue tend to act through perfusion. A perforation causes subarachnoid hemorrhage, and intracranial pressure rises abruptly. The monitored pathway is then threatened by reduced perfusion, not by dissection of the tissue around it. Perfusion failure need not begin with the loss of neural elements. It may begin with impaired, and potentially reversible, conduction (Lesnick et al., 1984; Hofmeijer and van Putten, 2012). Recent endovascular thrombectomy data are consistent with this. In a cohort with successful angiographic reperfusion, tree-based models separated functional outcome more consistently and efficiently by latency than by amplitude (Lee et al., 2026).

This is a difference of degree, not of kind. Both parameters carry information in every setting, and the question is how much weight each should carry. Where ischemia is the dominant threat, conduction is expected to slow before amplitude falls. Latency then deserves more weight than VEP practice now gives it. A second question follows. The conventional 10% latency threshold was calibrated for injury of any kind, and early conduction slowing may be smaller than that.

Before a parameter can be proposed as a serial intraoperative marker, its measurement must first be shown to be robust. A parameter that varies widely with recording conditions cannot be tracked reliably over time. The present study addresses this prerequisite for VEP. Our objective was to compare the within-eye spatial variability of P100 amplitude and latency across the standard occipital recording sites, and to evaluate whether latency possesses measurement characteristics that would justify its prospective investigation as a neuroendovascular monitoring parameter.

## 2 Two procedural injury environments

### 2.1 Direct surgical neural injury

Open surgery threatens the visual and sensory pathways in more than one way. Which way dominates depends on what the operation does. Where a lesion is separated from the surrounding brain, the threat is physical (Jashek-Ahmed et al., 2021; Oliva et al., 2023). Traction, compression, coagulation heat, retraction, and direct manipulation act on the tissue itself (Roca and Ramorino, 2023). The consequence follows a fairly direct sequence. Mechanical or thermal stress disturbs neuronal and synaptic function. The population of neurons that can be recruited by a stimulus falls. The remaining response loses synchrony (Calma et al., 2025). The summed cortical potential then shrinks in amplitude, and with more severe injury the waveform is lost (Ji et al., 2013).

Amplitude is a reasonable primary marker for this kind of injury. It is sensitive to the size of the activated neural population and to the synchrony of the response, and these are precisely the quantities that direct injury reduces (Calma et al., 2025). When the underlying threat removes or desynchronizes functioning neural elements, a fall in amplitude tracks the injury in a physiologically faithful way.

### 2.2 Perfusion-related endovascular injury

Neuroendovascular procedures place the same pathways at risk through a different primary event. The tissue is usually not touched. What changes first is blood flow. Arterial flow disturbance reduces the delivery of oxygen and glucose. Energy metabolism fails, and ionic gradients across the neuronal membrane begin to break down (Hofmeijer and van Putten, 2012). Before neurons are lost, conduction along the axon and its myelin can become impaired (Lesnick et al., 1984). Here the injury has a specific cellular basis. Oligodendrocytes maintain the myelin that supports rapid axonal conduction, and they are acutely vulnerable to ischemic stress (McDonald et al., 1998; Back et al., 2002). They sustain excitotoxic and oxidative injury at energy deficits that neurons can initially survive (Back et al., 2002). Conduction slowing may therefore precede neuronal death and waveform loss. Signals then arrive at the cortex late and less synchronously. Latency prolongation may appear first, and amplitude reduction or waveform loss may follow only as the insult deepens (Lesnick et al., 1984; Lee et al., 2026).

This sequence is proposed rather than established, but it points to a genuine difference in physiology. Direct surgical injury primarily threatens the structural and cellular integrity of neural tissue, whereas endovascular injury more often begins as a disturbance of energy-dependent conduction. The distinction is one of dominant mechanism, not of absolute category. Direct procedures can compromise perfusion, and endovascular procedures can injure tissue directly. What differs is which mechanism predominates, and therefore which measurement is best placed to register the earliest change.

## 3 Why amplitude-centered criteria may be insufficient in perfusion injury

The argument so far is not that amplitude is a poor marker. In severe injury it falls, and that fall reflects a real loss of functioning neural elements. The difficulty is specificity rather than sensitivity. The same factors that make amplitude responsive to injury also make it responsive to conditions that have nothing to do with the pathway. Recording geometry, stimulus quality, anesthetic depth, and signal averaging all change amplitude, and the size of that change can be as large as an early ischemic one (Hayashi and Kawaguchi, 2017; Šuštar Habjan et al., 2025).

This matters most at the beginning of an insult, which is exactly when a warning would be most useful. Early perfusion dysfunction is expected to be small in magnitude (Hofmeijer and van Putten, 2012). A small fall in amplitude, taken alone, cannot easily be separated from baseline variability or technical drift (Mattogno et al., 2023). The signal that one would most want to catch is the one that amplitude is least able to isolate. Amplitude may therefore be highly sensitive to severe injury but insufficiently specific for early perfusion-related dysfunction. This is the gap that motivates asking whether another feature of the same waveform behaves more stably, and it is the question the present data address.

## 4 Empirical measurement asymmetry in VEP

### 4.1 Study population and measurements

We first state how these measurements relate to the hypothesis. The comparison that follows is not a test of the central hypothesis, and it is not, by itself, evidence that latency detects ischemia. It is a spatial comparison across occipital electrodes recorded at the same instant, and it therefore addresses a prerequisite question: whether P100 latency can be measured reproducibly enough for serial, time-based monitoring. Accordingly, these measurements assess the feasibility of the proposed monitoring approach rather than the hypothesis itself. To examine this measurement question directly, we compared the two parameters in a clinical VEP dataset. The data came from a retrospective series of patients with available pattern-reversal VEP recordings. The study was approved by the Institutional Review Board of Inje University Haeundae Paik Hospital (IRB No. 2026–08-001), and the requirement for informed consent was waived. Recordings used a standard pattern-reversal checkerboard stimulus delivered monocularly, with active electrodes at O1, Oz, and O2 referenced to a midfrontal electrode and a bandpass of about 1–100 Hz. The P100 was taken as the major positive peak near 100 ms, and its latency (ms) and amplitude (μV) were measured at each site. An eye was eligible when both values were available at all three sites; when both eyes qualified, both were included.

The final set comprised 49 eyes from 28 patients, of whom 21 contributed both eyes and 7 contributed one. The median age was 69.0 years (IQR, 62.5–75.3), 20 patients (71.4%) were male, and all were right-handed. Anterior circulation ischemic stroke was the most common lesion category (46.4%), followed by retinal vascular lesions (17.9%), posterior circulation ischemic stroke (14.3%), multiple-territory ischemic stroke (14.3%), and hemorrhagic cerebrovascular lesions (7.1%) (Table 1).

**Table 1:** Demographic and clinical characteristics of the study population.

Characteristic	Total (N = 28)
Age, years	69.0 (62.5–75.3)
Sex, n (%)	
Male	20 (71.4)
Female	8 (28.6)
Handedness, n (%)	
Right-handed	28 (100.0)
Primary lesion category, n (%)	
Retinal vascular lesion	5 (17.9)
Anterior circulation ischemic stroke	13 (46.4)
Posterior circulation ischemic stroke	4 (14.3)
Multiple-territory ischemic stroke	4 (14.3)
Hemorrhagic cerebrovascular lesion	2 (7.1)

Values are presented as median (interquartile range) or number (%).

For each eye and each parameter, spatial variability was quantified with a spatial variability index (SVI), defined as the range of the parameter across O1, Oz, and O2 divided by its mean across the three sites and expressed as a percentage:


$$\mathrm{SVI}\;(\%)=\frac{\max(X_{O1},X_{\mathrm{Oz}},X_{O2})-\min(X_{O1},X_{\mathrm{Oz}},X_{O2})}{\text{mean}(X_{O1},X_{\mathrm{Oz}},X_{O2})}\times 100$$

Here the maximum, minimum, and mean are taken over the parameter’s values at O1, Oz, and O2, and X is either P100 latency or P100 amplitude. We selected the range normalized to the mean because it provides an intuitive, scale-independent measure of between-site variability without assuming any distributional form. A higher SVI indicates greater variation between sites, and an SVI near zero indicates consistency.

For each eye, the difference between the two parameters was taken as the SVI difference:


SVIdifference=amplitudeSVI−latencySVI

expressed in percentage points, so that a positive value indicates greater spatial variability in amplitude than in latency. The number and proportion of eyes and patients with a positive difference were recorded.

To display the relative spatial distribution of each parameter, the value at each site was normalized to the within-eye mean:


$$\text{normalized}\;X_{i}=\frac{X_{i}}{\text{mean}(X_{O1},X_{\mathrm{Oz}},X_{O2})},i=O1,\mathrm{Oz},O2$$

A normalized value of 1 equals the within-eye mean, and values above or below 1 indicate relative increases or decreases. Raw values at the three sites are shown as connected-line plots (Figure 1), and the normalized values as heatmaps (Figure 2).

**Figure 1:**
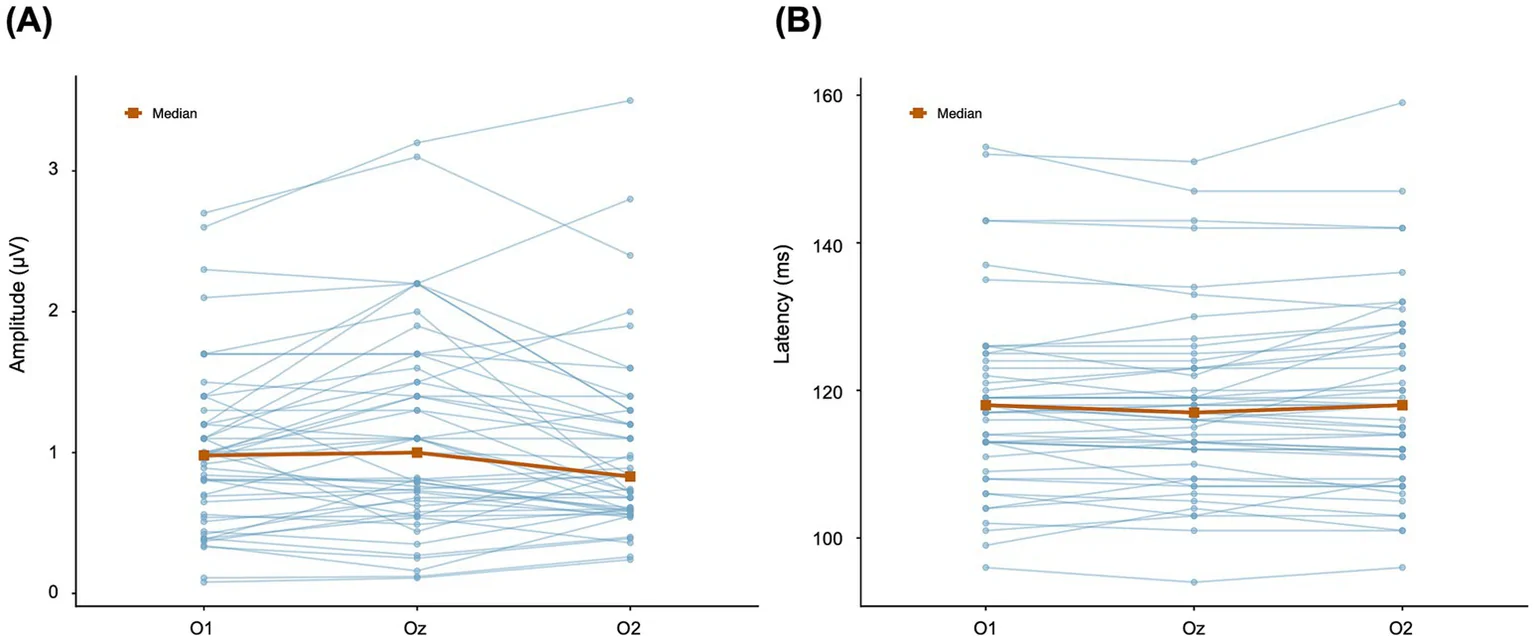
Eye-level distribution of P100 **(A)** amplitude and **(B)** latency across occipital electrode positions. Connected-line plots show raw P100 amplitude and latency measurements at O1, Oz, and O2 for each eligible eye. Light blue points and lines represent individual eyes, with measurements from the same eye connected across electrode positions. Orange squares and lines indicate the median value at each recording site.

**Figure 2:**
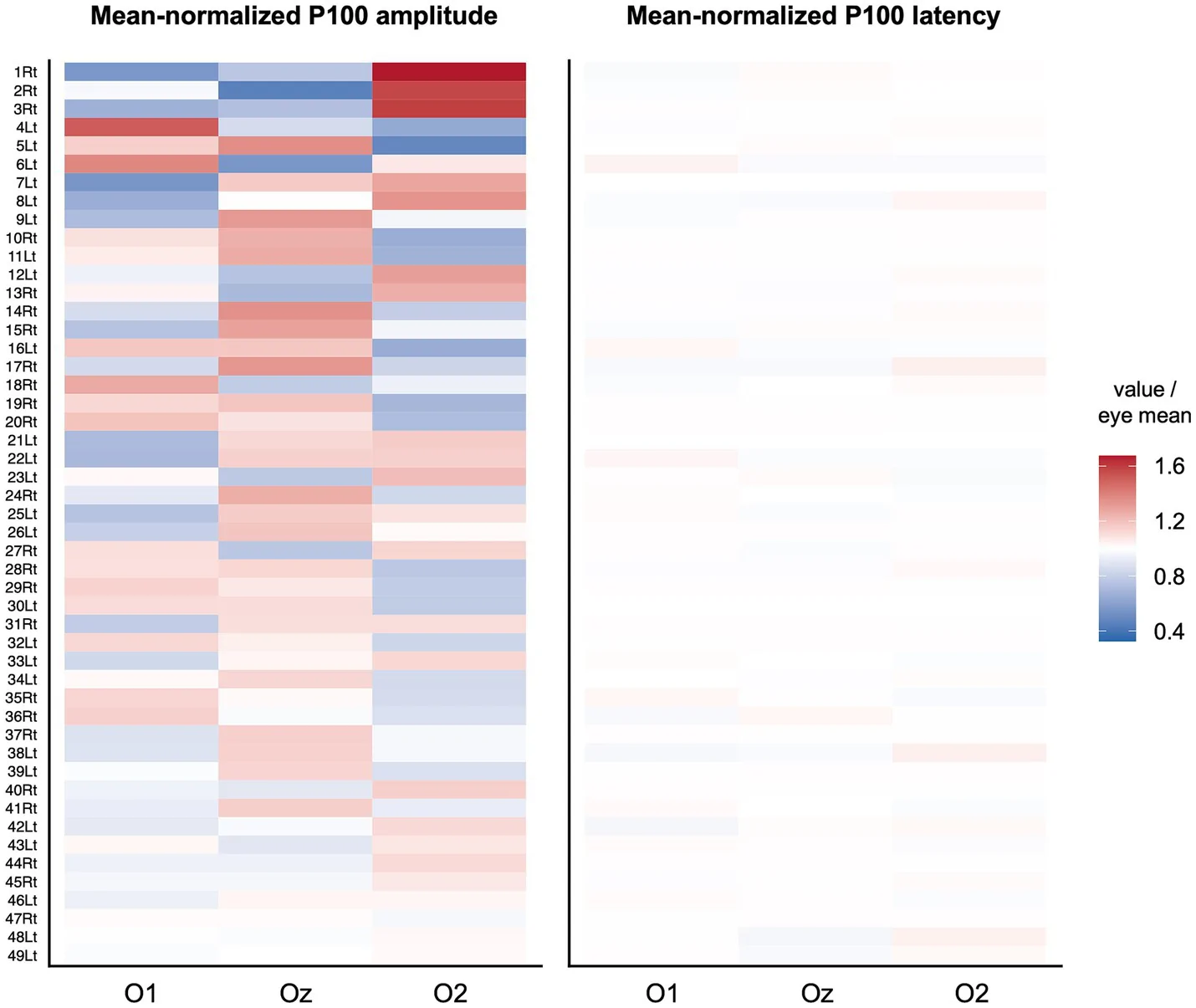
Eye-level spatial distribution of mean-normalized P100 amplitude and latency across occipital recording sites. Heatmaps show P100 amplitude and latency at O1, Oz, and O2 after normalization to the mean value across the three recording sites within each eye. Each row represents one eligible eye. Red indicates a value above the within-eye mean, blue indicates a value below the within-eye mean, and white indicates a value close to the within-eye mean. Greater color variation in the amplitude heatmap reflects broader spatial variability than that observed for latency.

Amplitude and latency SVIs were compared with paired Wilcoxon signed-rank tests at the eye and patient levels. For the patient-level analysis, the two eyes of a patient were averaged, and a 95% confidence interval for the median SVI difference was obtained by resampling patients 10,000 times. Analyses used R 4.5.2.

### 4.2 Findings

The two parameters behaved very differently. At the eye level, the median amplitude SVI was 40.9% (IQR, 26.5–55.1), whereas the median latency SVI was 2.10% (IQR, 0.89–3.69). Amplitude variability exceeded latency variability by a median of 38.4 percentage points, and this held in 47 of the 49 eyes (95.9%). The difference was significant on a paired comparison (Figure 3A, *p* < 0.001). The patient-level analysis gave the same result. The median amplitude SVI was 38.1% (IQR, 29.7–57.6) against a latency SVI of 2.29% (IQR, 1.44–3.08), with a median difference of 35.6 percentage points and higher amplitude SVI in all 28 patients (Figure 3B, 100%; bootstrap 95% CI, 29.4–51.7 percentage points).

**Figure 3:**
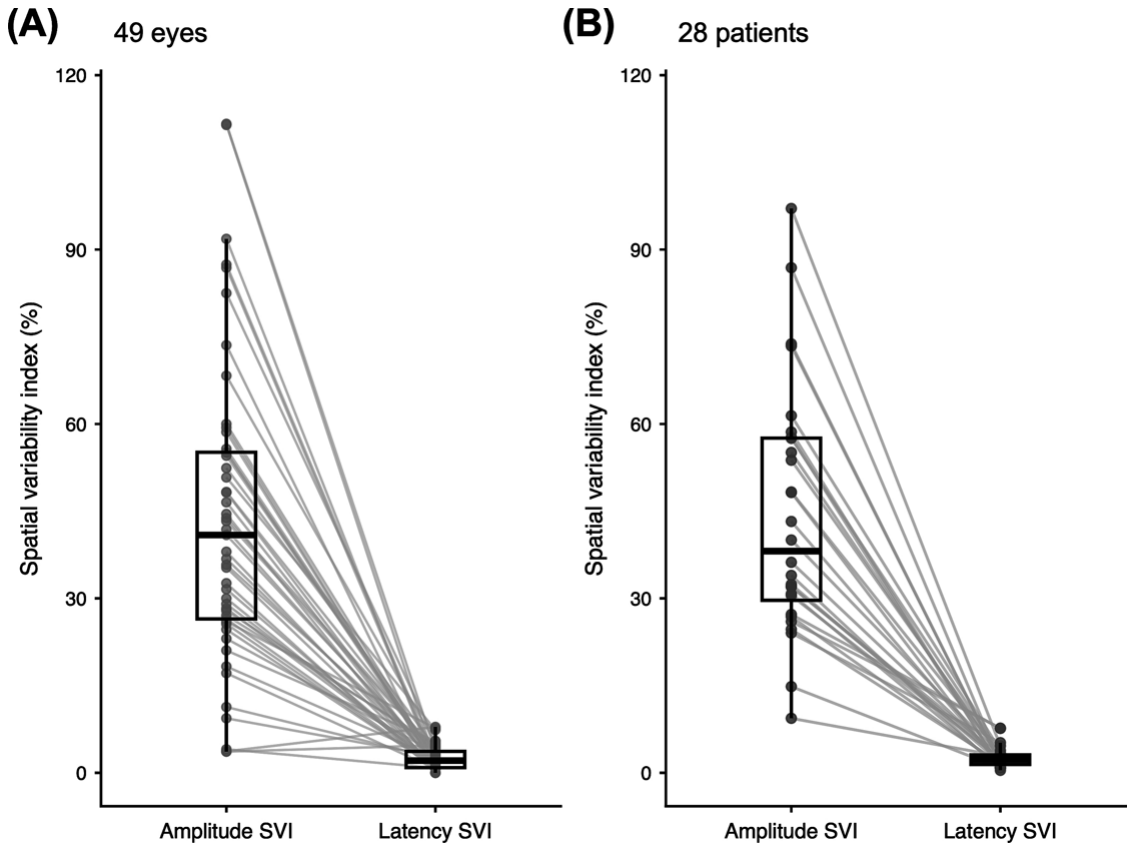
Comparison of P100 amplitude and latency spatial variability at the **(A)** eye level and **(B)** patient level. Gray dots represent individual eyes or patients, and gray lines connect paired amplitude and latency SVI values within the same eye or patient. Boxplots indicate the median and interquartile range, with whiskers extending to 1.5 times the interquartile range. SVI, spatial variability index.

The figures show the same pattern in raw form. In the connected-line plots, amplitude moved substantially across O1, Oz, and O2 within an eye, while latency stayed close to a single value across the three sites in most eyes (Figure 1). The mean-normalized heatmaps show the same contrast, with broad spatial variation in amplitude and a nearly uniform latency field (Figure 2).

### 4.3 Interpretation

These numbers describe two different measurement properties of one waveform. Amplitude depends on the size of the activated neural population, on the synchrony of the response, and on dipole geometry and volume conduction, all of which are sampled differently at each electrode. It therefore behaves as a spatially sampled cortical field measure. Latency depends instead on the time from stimulus to cortical response, and this arrival time changes little between adjacent occipital electrodes. It therefore behaves as a comparatively stable temporal measure of signal arrival.

One boundary should be stated plainly. These recordings compared three sites at the same moment. They did not track a single site over time. The data therefore speak to spatial robustness, not to temporal reproducibility during a procedure. And they do not show that latency detects ischemia. They show only that amplitude and latency carry different measurement properties, one strongly shaped by where the signal is recorded and the other much less so. The present results therefore establish a difference in spatial variability, and with it the measurement robustness of latency. They do not establish temporal stability during monitoring, and they do not establish superiority of latency for ischemia detection. Neither property is tested here.

## 5 Supportive evidence from endovascular SSEP

The VEP measurement asymmetry above shows that P100 latency is easier to measure consistently. It does not show that P100 latency changes carry clinical meaning during ischemia. To answer that question, a different kind of evidence is needed: latency measured during an actual ischemic event, alongside known clinical outcomes. As it happens, such evidence has recently become available for a closely related test, the somatosensory evoked potential (Lee et al., 2026). In that research, somatosensory evoked potentials from endovascular thrombectomy were examined against 90-day functional outcome (Lee et al., 2026). The study ran three separate tree-based analyses, and in each one latency, not amplitude, separated favorable from unfavorable outcomes (Lee et al., 2026). Max P40 latency was the strongest single discriminator, reaching an area under the curve near 0.89 (Lee et al., 2026). In the combined analysis it formed the first split, and a change in N20 latency formed the next (Lee et al., 2026). Amplitude variables were consistently outranked and did not form the splits, and the preference for latency held on bootstrap resampling (Lee et al., 2026).

Two features make this relevant here. The association held even in patients with successful angiographic reperfusion, so it did not simply track whether the vessel was open (Lee et al., 2026). And latency outperformed amplitude for the same outcome (Lee et al., 2026). Together these suggest that endovascular ischemic injury may contain a graded conduction-dysfunction phase that waveform presence or normalized amplitude change alone does not adequately represent (Lee et al., 2026).

These results come from SSEP, not VEP, so they do not prove that P100 latency behaves the same way. A further caution is specific to the visual system. SSEP and VEP do not share the same susceptibility to retinal and ophthalmic hypoperfusion. The extrapolation from SSEP latency to VEP latency must therefore be made conservatively. We treat the SSEP evidence as a proof of principle only. It shows that evoked potential latency can carry clinically meaningful information during ischemia. It is not direct evidence for VEP, which remains to be tested. Still, the two tests are built alike. Both begin with a sensory stimulus, carry the signal along a central pathway, and end in a cortical response with a measurable latency and amplitude. This shared structure is what makes the SSEP finding a reason to test latency in the visual system.

The three pieces of evidence do different jobs. The SSEP study suggests that latency reflects real ischemic dysfunction (Lesnick et al., 1984; Lee et al., 2026). The VEP data we presented above show that latency can be measured consistently. Whether VEP latency actually responds to visual pathway ischemia has not been tested, and that is the task for the next study.

## 6 A biological model: perfusion failure as a conduction disorder

If latency is worth watching during perfusion-related injury, there should be a mechanism that explains why. That mechanism can be stated as a simple model. Direct injury is, at its core, a question of how many neural elements remain and how well they fire together. Perfusion failure is a question of how well the surviving elements conduct. The two need not produce the same early signal.

When flow falls but has not yet stopped, neurons are not lost at once. What fails first is the energy-dependent machinery of conduction, and the oligodendrocyte sits at the center of it (Astrup et al., 1981; Hofmeijer and van Putten, 2012). Oligodendrocytes maintain the myelin that makes saltatory conduction fast, and they run on their own continuous energy supply (Nave, 2010). They are more vulnerable to ischemia than neurons, sustaining injury at energy deficits that neurons still survive (McDonald et al., 1998; Back et al., 2002). Within minutes of ATP depletion this machinery begins to fail in ways that slow conduction directly (Hofmeijer and van Putten, 2012). The myelin sheath takes on water and swells, and the sodium channels that should sit densely at the nodes of Ranvier drift out of position (Fern and Möller, 2000; Susuki, 2013; Hinman, 2014; Hinman et al., 2015; Coban et al., 2017; Han et al., 2017; Looser et al., 2024; Arafa et al., 2026). Saltatory conduction degrades, and conduction velocity falls while the neurons themselves are still firing (Nave, 2010). Because the myelin fails before the neurons, timing is disturbed before firing synchrony is, and if flow is restored in this window the change may reverse. If ischemia continues, calcium floods the oligodendrocyte, its processes retract from the axon, and membrane integrity is lost, carrying the tissue toward the structural damage that finally abolishes the waveform (Figure 4) (McDonald et al., 1998; Back et al., 2002).

**Figure 4:**
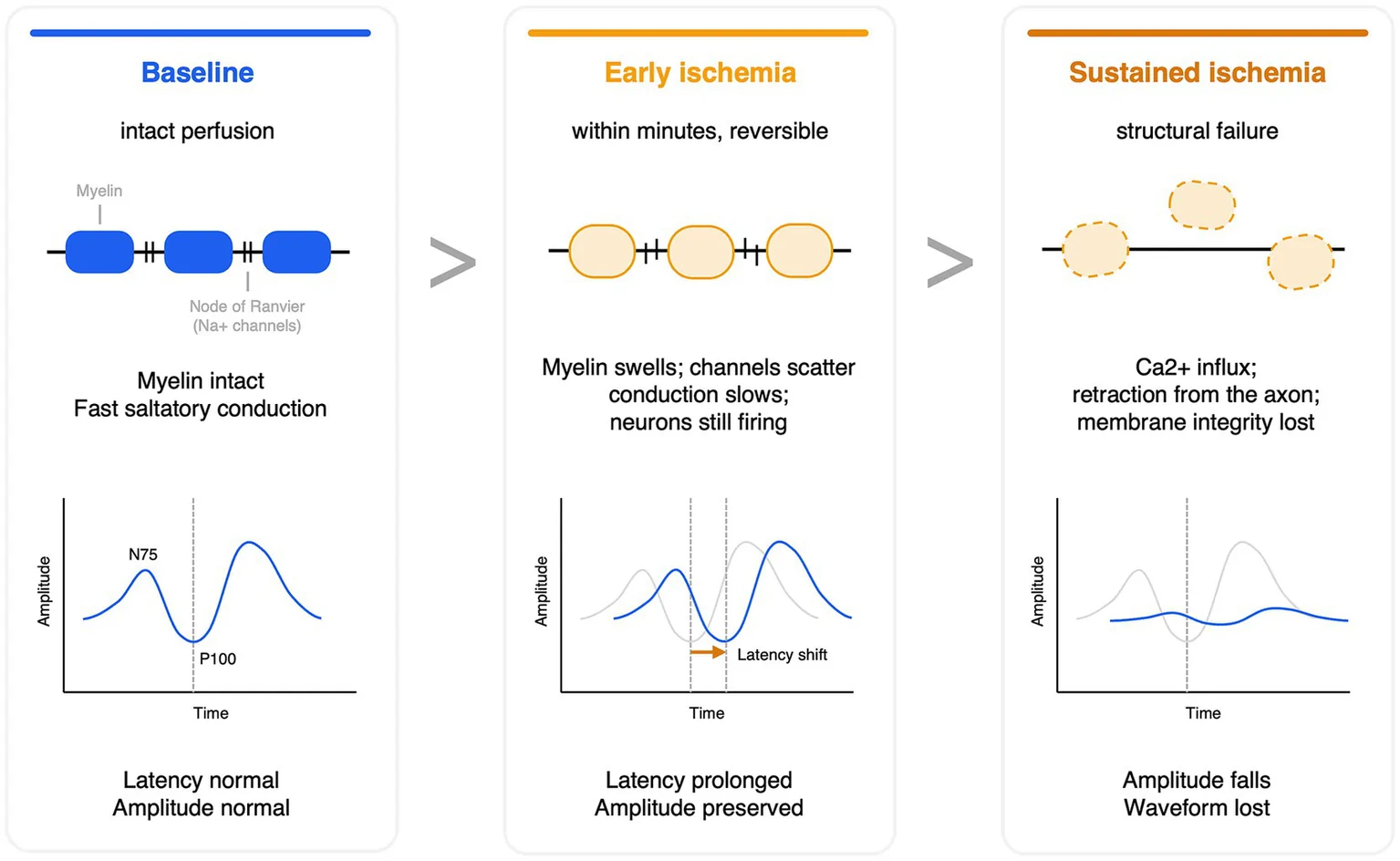
Proposed mechanism by which perfusion failure alters conduction before it abolishes the response. At baseline, intact myelin and clustered nodal sodium channels support fast saltatory conduction, and the evoked response has a normal latency and amplitude. In early ischemia, within minutes of ATP depletion, the myelin sheath swells and nodal sodium channels scatter; conduction slows while neurons continue to fire, so latency prolongs while amplitude is preserved, a change that may reverse if flow is restored. With sustained ischemia, calcium influx and process retraction cause loss of membrane integrity and the response collapses, with amplitude falling while latency remains prolonged. The dashed curve marks the baseline waveform for comparison. The diagram is schematic and not drawn to scale.

Although both paths can converge on the same endpoint, a small or absent waveform, they do not begin at the same point. Latency is of interest here because it reads the start of the perfusion path, the part that amplitude is slow to show.

The model should be read with caution. It describes a tendency, not a fixed order. The sequence is expected to vary with ischemic severity, pathway segment, collateral status, and duration. A severe or abrupt occlusion may collapse amplitude with little warning, while a milder or slower one may pass through a longer phase of conduction slowing. The individual mechanisms cited here come from peer-reviewed experimental work. What remains uncertain is the synthesis: assembling them into this clinical model is an interpretive step, and other readings are possible. Its value is not in fixing an order but in identifying timing as a signal worth watching early.

## 7 Central hypothesis

These sections converge on a set of claims. Amplitude and latency both carry information, and neither should be set aside. But VEP has been held back as a monitoring tool partly by one problem: P100 amplitude is so variable that a change in it is hard to read, and monitoring built around amplitude has struggled to be reliable. Neuroendovascular procedures invite a different view. Their essential threat is perfusion rather than direct manipulation, and perfusion injury should show first in conduction, that is, in latency. This is the setting in which VEP is worth reading with more weight on latency than on amplitude, and VEP allows it: P100 latency varies far less than amplitude, as the present data show, and can act as a reliable variable. We state the claims separately so that each can be tested.

*Primary hypothesis*. During neuroendovascular procedures that place the central visual pathway at perfusion risk, reproducible P100 latency prolongation may identify evolving conduction dysfunction before major amplitude loss or waveform disappearance.

This hypothesis generates two testable predictions:

- P100 latency will show sufficient within-procedure reproducibility to permit reliable serial tracking.
- Latency may serve as an early marker of conduction slowing during mild or reversible perfusion compromise, preceding or occurring without major amplitude reduction, whereas more severe or sustained injury may eventually produce abnormalities in both latency and amplitude.

The present data support the measurement rationale underlying the first prediction, but do not establish within-procedure reproducibility. Both predictions require prospective intraoperative validation, particularly because spatial robustness does not necessarily imply temporal reproducibility.

## 8 A proposed framework for reading VEP during a procedure

The hypothesis points to a practical way of reading serial VEP as a case unfolds. Because amplitude is the more variable channel, the reading leans on latency and weighs amplitude only when its change is large and lasting. What follows is a way of reading, not an alarm criterion, and it sets no threshold.

- Stage 0. Stable. A recognizable P100 remains present, with reproducible latency across repeated recordings. Amplitude may fluctuate without indicating injury.
- Stage 1. Possible early perfusion dysfunction. P100 latency becomes reproducibly prolonged while the waveform remains identifiable. Latency may capture early conduction slowing before a major amplitude reduction appears.
- Stage 2. Progressive dysfunction. Persistent latency prolongation is accompanied by a large, sustained amplitude decline and increasing waveform dispersion, suggesting more advanced functional compromise.
- Stage 3. Severe dysfunction or absent response. The P100 becomes markedly attenuated or disappears. This may follow earlier abnormalities or occur abruptly during a severe event. When waveform loss is discordant with the procedural context, non-ischemic causes should first be excluded, including deepening anesthesia, changes in pupil size or eye position, and failure of stimulus delivery.

The framework does not say how much latency change matters, or over what time, because the present data cannot set that. What it does is fit the way VEP actually behaves. It reads the stable parameter, latency, as the early signal, and it declines to over-read the unstable one, amplitude, until the change is unmistakable.

## 9 Appropriate clinical models and the retinal confound

This hypothesis is about one specific part of the visual system, and that decides which procedures can test it. It concerns the central visual pathway, the retrochiasmal segment that runs from the optic tract through the optic radiation to the occipital cortex. Any test of the hypothesis must therefore isolate that segment, using procedures that place the retrochiasmal pathway at perfusion risk while leaving the eye’s input intact. Otherwise the retina confounds the measurement. If it becomes ischemic, P100 latency can prolong because the signal entering the eye has been degraded, not because central conduction has slowed (Hayashi and Kawaguchi, 2017; Šuštar Habjan et al., 2025). In that case the two would look the same on the trace, and the hypothesis would be untestable. The three cohorts below are arranged accordingly. A preliminary cohort asks whether latency can be tracked over time at all. A primary cohort then tests the hypothesis in procedures where retinal input is intact. An exploratory cohort takes the cases where it is not.

### 9.1 Preliminary cohort: temporal stability of intraoperative P100 latency

Before either of the cohorts described below is opened, one prerequisite remains unmet. The data reported above establish spatial robustness, not temporal stability. The distinction matters. A parameter that is uniform across electrodes at one instant need not be uniform at one electrode across a procedure. A preliminary cohort should therefore establish that intraoperative P100 latency is stable enough over time. Early ischemia is expected to produce small changes, and the measurement must be able to resolve them. This study is deliberately simple. It can precede recruitment for the main cohorts, or run alongside them. In practice its data would accumulate first, because the ischemic events the primary cohort targets occur in only a small fraction of interventions.

Its design mirrors the analysis reported here, with time substituted for space. In procedures already monitored with VEP there is ordinarily a period during which the visual pathway is not at risk, typically before and during the access phase. Three to five or more consecutive recordings can be obtained from each eye during that period. For each eye and each parameter, a temporal variability index would be computed as the range across those recordings divided by their mean, expressed as a percentage. This is the spatial index of Section 4 with time in place of space. Amplitude and latency would then be compared with paired tests at the eye and patient levels, both at a single fixed electrode and across the montage. Anesthetic depth, stimulus delivery, and pupil size would be recorded alongside, so that technical drift can be separated from measurement noise.

The comparison itself is not what decides the matter. That is the repeatability coefficient for latency: the smallest change that can be distinguished from measurement noise in a pathway that is not at risk. This number sets the floor for any latency alarm, and it should be stated in advance as the criterion for proceeding. If it lies well below the conventional 10% criterion, serial latency tracking is feasible, and a lower threshold can be considered on evidence rather than assertion. If it approaches or exceeds 10%, the first prediction of the hypothesis fails at the outset. The proposal should then be abandoned in its present form rather than carried into the larger cohorts. Ten patients, or twenty eyes, would support an initial estimate, which should be reported with its precision.

### 9.2 Primary mechanistic cohort

The primary mechanistic cohort is drawn from procedures in which retrochiasmal perfusion is the dominant visual risk. These include posterior circulation thrombectomy, posterior cerebral artery branch interventions such as angioplasty, stenting, and aneurysm treatment, and embolization of occipital arteriovenous malformations or dural fistulas near the calcarine cortex and optic radiation. Each maps onto ischemia of the optic radiation or occipital cortex, and each produces a visual field outcome, and often a diffusion-weighted lesion, that can be matched against the recorded latency.

In the primary cohort, preservation of retinal input would not be assumed. It would be confirmed at the time of recording. A concurrent electroretinogram (ERG) showing an intact retinal response, together with documentation of patent ophthalmic arterial supply and an unremarkable retinal examination, would establish that a P100 latency change arises from retrochiasmal conduction rather than from degraded retinal input. Because the target of this hypothesis is posterior circulation, retrochiasmal ischemia, anterior circulation events that threaten the ophthalmic artery and retina fall outside its scope and are addressed separately in the exploratory cohort.

### 9.3 Secondary exploratory cohort

The secondary exploratory cohort is where the retinal confound lives, and it is kept apart on purpose. Ophthalmic artery aneurysm treatment and paraclinoid internal carotid flow diversion are the clearest examples. A visual change in these cases can arise from several sources at once: retinal ischemia, ophthalmic artery hypoperfusion, optic nerve ischemia, ischemia of the chiasm or pathway, mass effect from a thrombosing aneurysm, or an embolic retinal artery occlusion (Ota et al., 2012; Nowicki et al., 2020). Because a retinal cause would move P100 latency through the input rather than through central conduction, these procedures cannot test the hypothesis cleanly (Šuštar Habjan et al., 2025). They are still worth studying, but as a separate cohort in which electroretinography, an assessment of retinal perfusion, and an ophthalmologic examination are recorded alongside the VEP, so that the origin of any latency change can be located.

### 9.4 Territories requiring parallel monitoring

Even within the primary cohort, few territories are entirely clean. Anterior choroidal artery procedures place the visual pathway at risk but threaten the motor pathway as well, and are better monitored with motor evoked potentials in parallel. Interventions on the inferior division of the middle cerebral artery can involve language and other cortex, and call for electroencephalography and language monitoring alongside VEP. The posterior cerebral and calcarine territory remains the most direct single candidate, because there the visual pathway is the structure principally at risk.

Each stage can fail on its own terms. The order matters, because a negative preliminary cohort would make the two larger studies pointless.

## 10 Discussion

The present data establish one thing directly: across O1, Oz, and O2, P100 latency varies far less than amplitude. From this it is reasonable, though not proven, to infer that latency is better suited to repeated measurement when recording geometry is the main source of variation. That adjacent occipital electrodes yield similar peak latencies is expected on physiological grounds. The contribution here is not the direction of this contrast but its magnitude in real clinical recordings. A latency SVI of 2.10% against an amplitude SVI of 40.9% quantifies how much more suitable latency is as a candidate for reproducible measurement. This satisfies a necessary prerequisite that must be met before latency can be proposed for serial monitoring. Everything beyond that is hypothesis. We draw no clinical recommendation from these data. The clinical value of latency as a monitoring parameter is a question for the prospective study proposed below, not a conclusion of the present work. Whether serial P100 latency can detect perfusion-related dysfunction before amplitude is lost has not been shown here, and spatial stability is not the same as stability over time.

Several cautions follow. The contrast between mechanical and perfusion injury is one of dominant mechanism, not a strict division. Latency need not always move first, since a severe insult can reduce amplitude with little warning. A prolonged latency is not specific to myelin injury, because axonal slowing, synaptic delay, temporal dispersion, and even delayed retinal input can all produce it. The recordings here were pattern-reversal studies in awake patients, which do not translate directly to flash VEP under anesthesia, and the sample is small and clinically heterogeneous, so no threshold can be drawn from it.

A further boundary defines the scope within which latency can serve. Latency can be read only while an identifiable P100 waveform persists; once the response is markedly attenuated or lost, latency is no longer measurable, and monitoring necessarily falls back on amplitude reduction and waveform loss. Latency is therefore informative in the early-to-intermediate phase of evolving dysfunction rather than across the full spectrum of ischemic injury, and for this reason it should be regarded not as an alternative to amplitude-based monitoring but as a potential adjunctive early-warning marker that complements it. The present framework also sets no threshold for a clinically meaningful latency change, no duration criterion, and no basis for separating reversible from irreversible dysfunction, and it does not establish that latency adds information beyond conventional amplitude-based criteria. Prospective studies are therefore needed to define an appropriate latency threshold and duration criterion, determine its relationship with reversibility, and establish whether latency provides incremental information beyond conventional amplitude-based monitoring. Because ischemic injury in the endovascular setting is expected to slow conduction before it reduces amplitude, that study should also test whether a latency alarm threshold below the conventional 10% is warranted.

The hypothesis is testable, and it is worth saying what would support or defeat it. It predicts that latency change should track perfusion events in time, that it should precede amplitude loss in milder events, and that it should recover when flow is restored in cases that do well. It would be weakened, and in its strong form defeated, if latency changes bore no relation to perfusion events, if serial latency proved as unstable as amplitude over time, or if amplitude alone monitored these procedures as accurately as latency added to it.

A prospective study could settle this, and its design is set out in Section 9. It would record baseline and serial P100 latency and amplitude with a retinal control such as electroretinography, over multichannel occipital electrodes, timed against angiographic events, and would follow postoperative visual fields and diffusion-weighted lesions. The primary question is simple: whether documented compromise of a visual-pathway artery is followed by reproducible P100 latency prolongation that precedes, or occurs without, major amplitude loss.

Amplitude-based VEP criteria were built for a surgical world of direct injury. Neuroendovascular procedures threaten the visual pathway mainly through perfusion, and perfusion failure may slow conduction before it destroys the response. Because P100 latency is spatially stable, and because latency has already proven informative in endovascular SSEP, it is the parameter worth testing first, as an adjunct to rather than a replacement for amplitude-based criteria. The claim of this paper is narrow: not that latency detects ischemia, but that it is stable enough, and plausible enough, to deserve a prospective trial.

## 11 Conclusion

Three claims should be kept apart at the end of this argument. The first is established here. Across O1, Oz, and O2, P100 latency varies far less than P100 amplitude, 2.10% against 40.9%. Latency therefore meets the measurement prerequisite that any serially tracked parameter must meet. The second is supported but not proven. Perfusion failure impairs conduction before it destroys neural elements, and evoked potential latency can therefore carry information during ischemia that amplitude does not yet show. The endovascular SSEP data suggest as much. The third is hypothesis. During neuroendovascular procedures that place the retrochiasmal pathway at perfusion risk, reproducible P100 latency prolongation may identify evolving conduction dysfunction early enough to be useful. A latency alarm threshold below the conventional 10% may be warranted in that setting.

The third claim is testable, and the sequence that would test it is specified in Section 9. A preliminary cohort must first show that intraoperative P100 latency is temporally stable enough to resolve small changes, and must report the smallest change it can resolve. Without that number no threshold can be set, and the remaining work is premature. A primary cohort of retrochiasmal perfusion-risk procedures would come next, with concurrent electroretinography holding retinal input fixed. It would test whether latency prolongation tracks angiographic events, precedes major amplitude loss, and predicts visual field and diffusion-weighted outcome. An exploratory cohort of ophthalmic-territory procedures would address the cases the primary cohort must exclude. We do not propose that latency replace amplitude. We propose that in the one setting where ischemia is nearly the only threat, latency be measured, weighted, and tested as the earlier of the two signals.

## Data Availability

The raw data supporting the conclusions of this article will be made available by the authors, without undue reservation.
